# Effect of Feed Mass, Reactor Temperature, and Time on the Yield of Waste Polypropylene Pyrolysis Oil Produced via a Fixed-Bed Reactor

**DOI:** 10.3390/polym16101302

**Published:** 2024-05-07

**Authors:** Saša Papuga, Jelena Savković, Milica Djurdjevic, Stefano Vecchio Ciprioti

**Affiliations:** 1 Faculty of Technology, University of Banja Luka, 78000 Banja Luka, Bosnia and Herzegovina; jelena.savkovic@student.tf.unibl.org; 2 Faculty of Mechanical Engineering, University of Banja Luka, 78000 Banja Luka, Bosnia and Herzegovina; milica.preradovic@student.mf.unibl.org; 3 Department of Basic and Applied Science for Engineering, Sapienza University of Rome, I-00161 Rome, Italy

**Keywords:** polypropylene, plastic waste, pyrolysis, fixed-bed reactor, response surface methodology

## Abstract

This paper presents the results of investigations into the pyrolysis of waste polypropylene in a laboratory fixed-bed batch reactor. The experiments were designed and verified in such a way as to allow the application of the response surface methodology (RSM) in the development of an empirical mathematical model that quantifies the impacts mentioned above. The influence of the mass of the raw material (50, 100, and 150 g) together with the reactor temperature (450, 475, and 500 °C) and the reaction time (45, 50 and 75 min) was examined. It has been shown that the mass of the raw material, i.e., the filling volume of the reactor, has a significant influence on the pyrolysis oil yield. This influence exceeds the influence of reactor temperature and reaction time. This was explained by observing the temperature change inside the reactor at three different spots at the bottom, middle, and top of the reactor. The recorded temperature diagrams show that, with greater masses of feedstock, local overheating occurs in the middle part of the reactor, which leads to the overcracking of volatile products and, from there, to an increased formation of non-condensable gases, i.e., a reduced yield of pyrolytic oil.

## 1. Introduction

Plastic waste can be converted into different fuels or chemicals in order to generate value and, therefore, reduce environmental pollution. Pyrolysis has gained a lot attention in scientific and industrial branches as a promising technique for converting plastic waste into more valuable products, which gain economic value. However, it is still challenging to find an efficient pyrolysis process for commercial applications [1]. Pyrolysis is a thermal degradation process that takes place at temperatures between 300 and 900 °C in an inert atmosphere, with liquid oil as the main product [2]. Polyolefin plastics decompose into heterogeneous products by a random-chain scission mechanism [3,4]. So, a wide range of products is produced in such a way, mostly linear paraffins and olefins. On the other hand, plastics, which have a high viscosity and a low heat transfer, produce more waxy products that need further upgrading [5,6]. The most applied catalysts are zeolite-based catalysts [7], silica-alumina-based catalysts [8], clay-based catalysts [9], and MCM-type mesoporous materials [6,10]. The employment of catalysts reduces both the reaction temperature and the time, as well as lowers the activation energy of degradation (pyrolysis) and the boiling temperature. Therefore, the energy consumption is reduced, and narrow-ranged hydrocarbons are formed [5,11]. The catalyst promotes the formation of lighter fractions in the liquid products, like gasoline, and reduces the process energy inputs [12].

The most common plastic waste streams contain HDPE, LDPE, PP, PS, PVC, and PET [5,13].

Some studies have investigated the pyrolysis of PP [14,15,16] and concluded that higher temperatures promote higher oil yields, while others [17,18,19] have studied PP pyrolysis in the presence of catalysts. However, it has been confirmed that a temperature higher than 500 °C leads to a decrease in pyrolysis oil [20].

The type of reactor, the residence time, the temperature, the pressure, the experimental conditions, and the feedstock are the most important factors that influence the pyrolysis process. Therefore, it is important to examine the limitations of the unit used in order to understand the most influencing parameters in this process [11]. The influence of reactor temperature or, as it is often presented, the reaction temperature on the yield and quality of the pyrolysis products of various plastic materials has been relatively well investigated [21,22,23,24,25]. However, by analyzing the published results, a significant variation in oil yield can be observed, even at the same or similar reaction temperatures, because each pyrolysis reactor or pyrolysis plant has different configurations, which influence the process’ products. Therefore, the impact of temperature on the distribution of pyrolysis products needs to be examined alongside other key process parameters, including the carrier gas flow and the duration of the reaction, the type of plastic used, the composition of the plastic mix, and the effects due to the presence of a catalyst, in order to evaluate their effect on the oil yield and its quality [3,26,27,28,29].

Certainly, this result can be prescribed by different reactor designs, but what is missing and at the same time worth noting is that, even with the same reactor designs, significant variations are possible in both the yield and quality of pyrolytic oil. In this study, it was assumed that different levels of reactor occupancy, expressed as the mass of raw material, will lead to pronounced non-uniformity of the temperature field inside the reactor, i.e., local overheating, which will lead to different yields and qualities of pyrolytic oil. Namely, the automatic temperature control of the reactor, trying to achieve the desired temperature in the layer of a raw material at the bottom of the reactor, performs overheating in the upper parts of the reactor, with a fixed layer [23]. Namely, when the plastic melts, it descends towards the bottom of the reactor, leaving the middle part of the reactor free, thus causing overheating of this part in comparison with the bottom part of the reactor. The upper parts of the reactor are occupied by a vapor phase, which, in this case, is overheated, leading to overcracking. In order to examine the aforementioned assumptions to mathematically describe and quantify each of them, the Box–Behnken design was used as a specific type of response surface methodology (RSM). RSM is commonly applied in the description of various processes [30,31,32,33], especially in the description of the pyrolysis of plastics [34,35,36,37,38,39,40,41,42,43,44,45,46].

## 2. Materials and Methods

### 2.1. Experimental Setup

**Figure 1:**
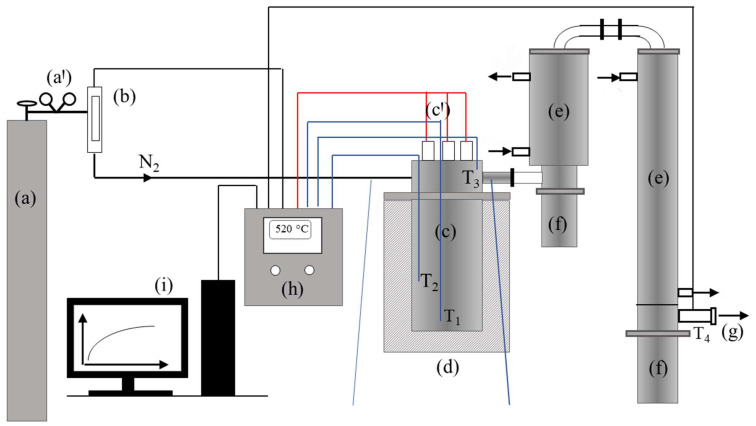
Block diagram of the experimental pyrolysis fixed-bed reactor: (a) cylinder with nitrogen gas; (b) mass gas flow meter; (c) pyrolysis reactor vessel; (d) thermal insulation; (e) steam condensation system; (f) separation system, i.e., the vessels for receiving the condensate; (g) discharge of non-condensable gas into the gas-washing system; (h) control box with the regulation system; and (i) PC [21].

Pyrolysis was performed in a laboratory-scale fixed-bed reactor, presented in Figure 1. A washed and shredded polypropylene waste sample was placed in the reaction vessel (c), occupying approximately the following volumes:

- 25% of the reactor’s effective volume for 50 g feedstock mass;
- 50% of the reactor’s effective volume for 100 g feedstock mass;
- 75% of the reactor’s effective volume for 150 g feedstock mass.

The average particle diameter of the shredded waste, determined by granulometry, was 1.63 mm.

Nitrogen served as the inert gas (a). Gas flow was regulated using a gas flow meter (b) set at 100 ncm^3^ min^−1^, and, after 10 min, an inert atmosphere was established. A PC (i) controlled the system using CelciuX (EJ1N-TC4A-QQ) (Omron, Kyoto, Japan) for thermal regulation, with CX-Thermo (Omron) managing the automatic control and system (h) by activating or deactivating the electric heater system (c) in accordance with the control loop [23,26,47,48]. The T1 thermocouple was responsible for measuring the temperature at the bottom of the reactor and acted as a control sensor for the electric heaters’ control loop, serving as a control variable. The set points for the reactor’s temperature were 450, 475, and 500 °C. The duration for which the feedstock remained at a set point temperature was defined as the reaction time. Once the designated reaction time elapsed, the regulation system was deactivated. Once the reactor temperature dropped below 100 °C, the reactor was disassembled, and the reaction vessel was separated from it (c). The reaction vessel’s weight was recorded both before and after the pyrolysis reaction to ascertain the mass difference, indicative of the solid residue produced. Additionally, the vessels collecting condensate (f) were disconnected from the condensation system (e) and weighed to determine the mass of condensable products, accounting for any condensate adhering to the internal walls of the condensation system (e) [25]. The reactor vessel took the form of a vertical cylinder, measuring 101.6 × 2 mm in size. It comprised a body (lower part) and a body cover (upper part), joined by a bolted flange joint with gaskets. The body was 200 mm high and served as the reaction vessel, while the upper part functioned as a dosing system, separated from the body. The design facilitated the simple weighing of the raw material and solid residue after the process finished, simplifying the setup of the material. A secondary cylindrical container, serving as a cover, was attached to the upper side of the reactor body, extending for a total length of 40 mm. This cover accommodated three K-type thermocouples and three stainless steel tubes (A304), each containing cartridge electric heaters with a combined power of 3 × 350 W. Upon attaching the cover to the reactor body, the electric heaters and thermocouples were positioned within the reactor body. The heaters extend to the bottom of the reactor body, while the three thermocouples were arranged at various heights within the reactor body, as shown in Figure 1. The thermocouples T1 (CH1) and T2 (CH2) were positioned at distances of 7 mm and 90 mm from the bottom of the reactor body, respectively. Constructed from stainless steel (A304), the reactor featured a 3 cm thick layer of stone wool thermal insulation applied to its outer wall. The temperature changes within the reactor were monitored at two distinct points. All the temperatures measurements were conducted using by K-type thermocouples and recorded using the CX-Thermo software package, Design Expert version 11 (Omron, Kyoto, Japan). Temperature regulation of the heater’s operation, i.e., temperature control, was carried out using the temperature controller CelciuX (OMRON, Japan). Prior to the commencement of the reaction, the adjustment of the PID (Proportional-Integral-Derivative) constants was conducted. Furthermore, the specified constants and other characteristic values within the regulation system were configured using the software. The flow of inert gas was measured and controlled by a mass flow meter/regulator (MASS VIEW model MV-304 Mass Flow Regulator, Bronkhorst High-Tech BV, Ruurlo, The Nederland) with a measurement range of 0.04 to 20 dm^3^ min^−1^, along with the capability for fine flow regulation. Nitrogen of 99.99% purity was used as a carrier gas.

### 2.2. Design of an Experiment and Mathematical Modeling

The design of an experiment and the development and assessment of mathematical models were performed using the BBD (Box–Behnken) type of response surface methodology (RSM), with support of the Deign-Expert 11 software (Stat-Ease, Inc., Minneapolis, MN, USA). BBD is a type of second-order design based on three-level incomplete factorial designs. The following variables were used as independent variables: reactor temperature (Factor 1), feedstock mass (Factor 2), and reaction time (Factor 3). Liquid yield, i.e., pyrolytic oil yield (Response 1), and rector solid residue (Response 2) were observed as the dependent variables. Table 1 presents the design of the experiments with the corresponding response measurement results. According to the used experimental design, a total of 16 experiments (design points) were carried out: i.e., 4 repetitions of the central point (475 °C; 100 g; 60 min) and 12 factorial points. This type of experimental design has proven to be reliable in testing the hypothesis of a complex interaction of 3 independent variables with a minimal number of experiments. A BBD design of this experiment, but with different independent variables and waste plastic mixture as the feedstock, was recently described [39]. Moreover, Ore and Adebiyi [40] chose a similar experimental design and a related RSM in studying the effects of temperature, sample weight, and reaction time on the yield of non-condensable gases in the pyrolysis of waste tire in a fixed-bed reactor.

The models were developed by fitting the numerical values of Response 1 and Response 2 into the corresponding equations, using the method of the least squares.

The general form of a second-degree polynomial is

(1) $$Y_{i}=\beta_{0}+{\beta_{1}X}_{1}+{\beta_{2}X}_{2}+{\beta_{3}X}_{3}+{\beta_{12}X}_{1}X_{2}+{\beta_{13}X}_{1}X_{3}+{\beta_{23}X}_{2}X_{3}+\;+{\;\beta }_{11}X_{1}^{2}+\beta_{22}X_{2}^{2}+\beta_{33}X_{3}^{2}+{{+\beta }_{12}X}_{1}X_{2}+e\;\;\;\;\;\;\;$$

where *Y_i_* is the response (Response 2); *X_i_* refers to the dependent variables (A, B, C); *β*_0_ is the constant coefficient; *β*_1_, *β*_2_, and *β*_3_ are the linear coefficients; *β*_12_, *β*_13_, and *β*_23_ are the coefficients of the interaction between the variables; *β*_11_, *β*_22_, and *β*_33_ are the quadratic coefficients; and e is the model error.

By selecting the BBD design, it was possible to test linear two-factor interaction (2FI) and quadratic equations, while cubic and higher-order equations in general were aliased, i.e., there were not enough unique design points to estimate all the model coefficients accurately, which led to contour plots displaying misleading shapes. Fit summary procedures (Table 2) collected preliminary statistics about the tested polynomial models, which were used to identify the starting point for the final model, i.e., the model for further in-depth study. A preliminary selection of the tested models was carried out via the Whitcomb Score, which uses the Sum-of-Squares *p*-value, the Lack-of-Fit *p*-values, and the Adjusted and Predicted R-squared as the parameters in a heuristic scoring system. The software labeled as “Suggested” the full-order model that met the criteria specified by the Whitcomb Score. It was found that only the quadratic model describing the change in the pyrolytic oil yield (Response 1) and the linear model describing the mass of solid residue (Response 2) could give the significant values of all the mentioned statistical parameters. Thus, the development and statistical proofing of these models are further described in this paper.

The statistical analysis of the developed mathematical models, including the determination of their statistical significance, was carried out using an analysis of variance (ANOVA), specifically applying Fisher’s test (F-test), with a significance level (alpha) of 0.05.

The analysis of variance assessed the significance of each model parameter’s effect on the variance in the outcome, relative to the total variance in all the observed model parameters.

## 3. Results

**Figure 2:**
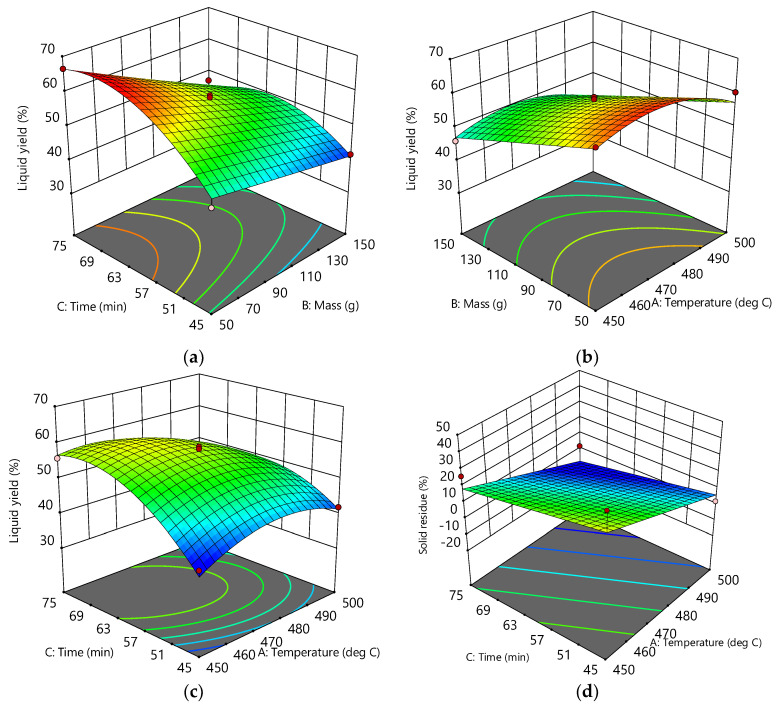
Response surface model plots and contours showing the following: (**a**) influence of the feed mass and time at 475 °C on the liquid yield; (**b**) influence of the feed mass and temperature at 60 min on the liquid yield; (**c**) influence of the time and temperature at 100 g feed mass on the liquid yield; and (**d**) influence of the time and temperature on the solid residue.

**Figure 3:**
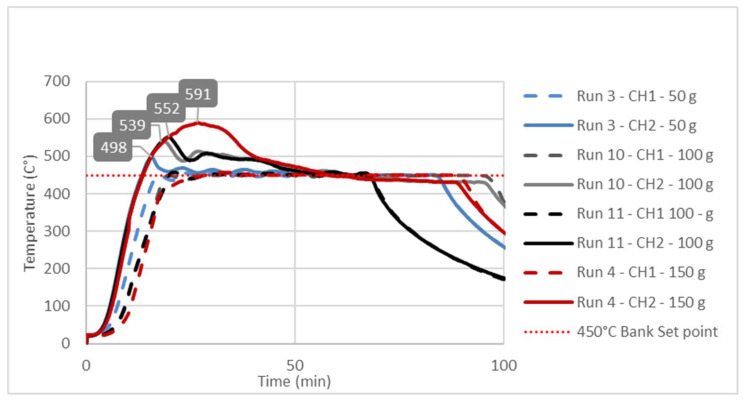
Temperature changes in the bottom (CH1 trend lines) and middle parts of the fixed-bed reactor during the pyrolysis process at a 450 °C preset bank temperature.

**Figure 4:**
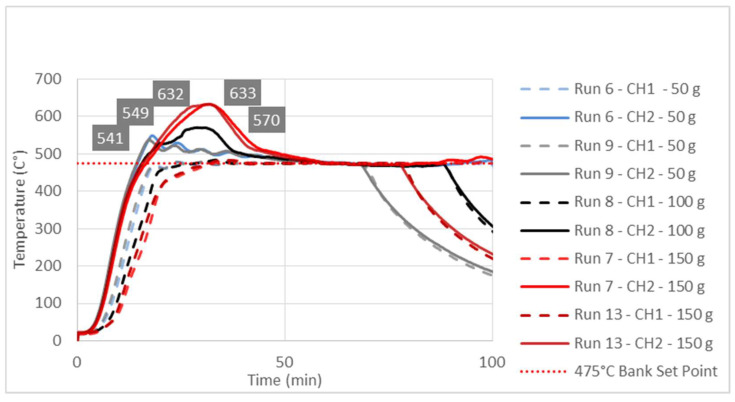
Temperature changes in the bottom (CH1 trend lines) and middle parts of the fixed-bed reactor during the pyrolysis process at a 475 °C preset bank temperature.

**Figure 5:**
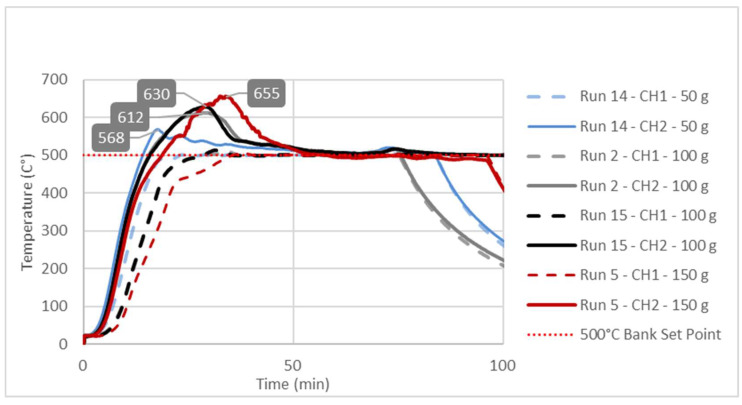
Temperature changes in the bottom (CH1 trend lines) and middle parts of the fixed-bed reactor during the pyrolysis process at a 500 °C preset bank temperature.

The effects of the independent variables (A, B, C) on the liquid yield (Response 1) and the reactor solid residue (Response 2) were examined using the numerical values shown in Table 1. As far as the liquid yield was concerned, it could be seen that these effects were not linear and that there were simultaneous interactions of several factors. For example, the highest liquid yield (66.54%) was obtained in run 6 (475 °C; 50 g; 60 min), which was slightly higher than the liquid yield (62.22%) obtained in run 3 (450 °C; 50 g; 60 min). However, one should keep in the mind that, during the occurrence of run 3, the conversion in the reactor was incomplete due to the very high amounts of solid residue in the reactor, mainly consisting of unreacted plastic. On the other hand, Table 1 shows that, even with the same temperature and reaction time and a quite similar solid residue, i.e., similar conversion of the reactor feed, significantly different liquid yields were obtained, only as a consequence of different feedstock masses. The most obvious examples of the former claim could be drawn from a simple pairwise comparison of run 3 vs. 4 or from a pairwise comparison of run 5 vs. 14 (Table 1). Such a complex nature of the interaction of dependent variables justified the application of RSM in the description of the mentioned phenomena. These interactions and their effects on liquid yield were described by a second-order polynomial (Equations (2) and (4)) and their related response surfaces (Figure 2) and, finally, explained by temperature diagrams (Figure 3, Figure 4 and Figure 5).

As for the effects of the dependent variables on Response 2 (solid residual), the behavior of the system was clearer and expected, i.e., a higher temperature and a longer reaction time promoted a smaller amount of solid residue (a higher reactor conversion rate). Therefore, such influences were described by a linear model (Equations (3) and (5)), without interaction factors (AB, AC, BC). In general, such a model could be developed with a less complex experimental design.

### 3.1. Model Development

By fitting the numerical values of Response 1 and Response 2 in the polynomials suggested in Table 2, the following empirical models were developed in terms of the actual factors:

Liquid Yield = −2339.348 + 8.889 × T + 0.128 × m + 10.041 × t − 0.011 × T × t − 0.005·m × t − 0.009 × T^2^ − 0.033 · t^2^ (2)

Solid Residue = +297.538 − 0.539 × T − 0.484 × t(3)

where T refers to the temperature (in °C), m refers to the feedstock mass (in g), and t refers to the reaction time (in min). These equations can be used to make predictions about the response for given levels of each factor. Here, the levels should be specified in the original units for each factor. These equations should not be employed to ascertain the relative impact of each factor because the coefficients are adjusted to suit the units of each factor, and the intercept does not align with the center of the design space.

In order to compare the relative contributions of the independent variables to the dependent variables (Responses 1 and 2), it is convenient to express the independent variables in a coded form. The coding law is given in Table 3.

By fitting the numerical values of Response 1 and Response 2 into Equation (1), i.e., the suggested equations from Table 2, and expressing the independent variables (A, B, C) in the coded form, the following empirical models were developed:

Liquid Yield = 57.51 − 2.55A − 7.62B + 5.09C − 0.6625AB − 4.17AC − 3.52BC − 5.48A^2^ + 0.4063B^2^ − 7.41C^2^ (4)

Solid Residue = 12.32 − 13.48 × A − 7.26 × C(5)

An equation formulated in terms of coded factors is applicable for predicting the response based on specific levels of each factor. Typically, high levels of the factors are coded as +1, while low levels are coded as −1. Utilizing a coded equation facilitates the assessment of the relative impact of the factors by comparing their coefficients.

According to Equation (4), i.e., the corresponding coefficients of factors A, B, and C, it is obvious that the influence of factor B far exceeds factors A and C. This means that an increase in the reactor feed mass (B) leads to a relatively lower liquid yield than that caused by an increase in temperature (A) or a reduction in the reaction time (C). Usually, such behavior is not expected. It is common sense to just leave enough time to allow the complete conversion of a given mass of raw material at the chosen temperature or to study temperature and time influences on some response of interests, which is common practice in many published studies [21,31,37,44].

The above statement is also proven by Equation (5), from which it can be seen that a simple increase in temperature and reaction time leads to a decrease in the solid residue and, finally, to a minimal solid residue, i.e., a complete conversion of the raw material. In other words, the mass of the feedstock in the reactor does not affect the total conversion of the reactor at a given temperature and time, which excludes the possibility that the conversion itself affects the liquid yield. This is consistent with the conclusion that can be drawn from the previously described pairwise comparisons (runs 3, 4, 5, and 14).

A fairly similar effect of feedstock mass on the yield of non-condensable gases in a fixed-bed reactor was observed in a tire waste pyrolysis study [40]. A similar empirical model was developed and statistically confirmed. However, no further explanation of such a behavior was given.

#### Statistical Testing

The developed models and all their coefficients were further proven by ANOVA (Table 4) and general-fit statistics (Table 5). Table 4 gives the results of the analysis of variance (ANOVA) regarding the developed empirical models (Equations (4) and (5)), determining the significance of the impact of each model parameter (A, B, C, AB, AC, BC, A2, B2) on the variance in the results.

Based on the values of the statistical parameters shown in Table 4, one could conclude that the quadratic model (Equation (4)) and the multi-linear model (Equation (5)) were reliable in describing the relative influence of A, B, and C on Response 1 and A and C on Response 2, respectively. Namely, the model F-value of 33.79 in the case of Response 1 and the model F-value of 13.7 in the case of Response 2 implied that the developed models were significant. There was only a 0.01% chance for Response 1 and a 0.07% chance for Response 2 that the F-values could occur due to noise. In general, *p*-values less than 0.0500 indicate that a model’s terms are significant. In the case of Response 1, A, B, C, AC, BC, A^2^, and C^2^ were the significant terms, while, in the case of Response 2, these were A and C. Insignificant model terms (AB and B^2^) with respect to Rresponse 1 are not shown in the Table 4. Generally, values greater than 0.1000 indicate that the model terms are not significant. The selection of the quadratic model for Response 1 is also supported by the Lack ofFit test, according to which there was only a 14.77% % chance that such a large F-value of the Lack-of-Fit test due to noise could occur.

Furthermore, the values listed in Table 5 support the significance of the selected models. Concerning Response 1, the Predicted R^2^ of 0.7716 was in reasonable agreement with the Adjusted R^2^ of 0.9387; i.e., the difference was less than 0.2. As far as Response 2 was concerned, the Predicted R^2^ of 0.4514 was in reasonable agreement with the Adjusted R^2^ of 0.6186; i.e., the difference was less than 0.2. The Parameter Adeq Precision measures the signal-to-noise ratio, with a ratio greater than 4 being desirable. In both cases, the values of these parameter indicated an adequate signal. Thus, one could conclude that the developed models were reliable and could be used.

### 3.2. Response Surfaces and Contour Plots

Graphical interpretations of the developed models are shown in Figure 2. As it can been seen from the plots (a), (b), and (c), the combined influence of the process variables, i.e., factors A, B, and C, and their interaction performs a curvature of the plots’ surface. This clearly indicates the existence of a complex interaction among these factors. In all three diagrams and their associated contours, the regions of the surfaces corresponding to the maximum liquid yield can be found. For example, from plot (a), it can be seen that, at a given temperature (475 °C), this area is in the range of 50–70 g of raw material mass, while time is in the range of 57–75 min. Using the development equations, this yield can be further optimized by finding the best combination of factors to reach the maximum liquid yield.

On the other hand, the surface of the solid residue’s response graph is flat or somewhat expected, which indicates that either the temperature or the time did not affect the total feed conversion in the reactor, that is, one of them had no influence on the amount of solid residue. Alternatively, as has already been emphasized, a simple increase in the temperature and reaction time led to a decrease in the solid residue and, finally, to a minimum solid residue.

### 3.3. Study of Temperature Diagrams

The observed significant effect of factor B or feedstock mass on Response 1, i.e., the liquid yield, could be explained by studying the temperature changes at characteristic points inside the reactor during the pyrolysis process. The temperature diagrams in Figure 3, Figure 4 and Figure 5 show simultaneous temperature changes at the bottom of the reactor (CH1) and in the middle part of the reactor (CH2), during the pyrolysis process, at the preset bank temperatures of 450, 475, and 500 °C, respectively.

From all three diagrams, it can be clearly observed that, in bottom part of the reactor (dashed lines corresponding to the CH1 temperatures), the desired temperature is almost ideally reached. Certainly, this is the consequence of the suitable PID temperature control loop, based on the temperature probe located at the bottom of the reactor. On the other hand, in all the cases, the temperatures in the middle part of the reactor, whose trend lines correspond to the CH2 temperatures, far exceed the preset temperatures (bank set points). This excessive exceeding of the preset temperature is more evident as the feedstock mass in the reactor grows. In all three diagrams, the highest excessive exceeding temperatures are observed during the experiments with the reactor loaded with 150 g of feedstock, while the lowest excessive exceeding temperatures are observed in those experiments with minimum reactor loading, i.e., 50 g of feedstock mass. Namely, the temperature control loop, trying to achieve the desired temperature in the layer of the raw material at the bottom of the reactor, causes overheating in the upper parts of the reactor with a feedstock fixed layer [23]. The upper parts of the reactor are occupied by the vapor phase, which, in this case, is overheated, leading to its overcracking, consequently reducing the pyrolysis oil yield. The temperature profile and the related heat transfer are decisive factors that determine the effective reactor performance, with thermal cracking being the most important step in producing pyrolysis oil [49,50,51].

Since the most heat-demanding (endothermic) process takes place in the lower part of the reactor, the heating and evaporation of molten plastic are common practices to monitor and control the temperature in that part. However, this leads to overheating and the excessive cracking of the steam in the upper sections occupied only by the pyrolysis vapors, as seen in Figure 3, Figure 4 and Figure 5. By considering all these aspects, if the temperature in the middle section of the fixed-bed reactor is not monitored, different yields of the liquid for apparently the same temperature, i.e., the temperature at the bottom of the reactor, are achieved. In general, such a practice leads to uncertainty in the results, especially compared to other studies, which usually involve different masses of the raw material, a different filling volume of the reactor, and, in general, different reactor settings.

As this study demonstrates, complex temperature changes can occur in the vapor phase only as a consequence of the mass of the raw material, i.e., the different reactor occupancy and the related non-uniformity of the temperature field in the fixed-bed reactor. In order to achieve a uniform temperature field within a fixed-bed reactor and, thus, a more consistent and comparable liquid yield, it is of utmost importance to develop an even more complex and precise temperature control system. This temperature control system should consist of at least two independent temperature control loops, one located close to the bottom and one close to the middle part of the reactor.

## 4. Conclusions

The effects of feedstock mass, temperature, and time were analyzed during the occurrence of a plastic pyrolysis process in a fixed-bed reactor in terms of liquid yield.

By means of RSM, mathematical models that described these effects were developed. The significance of the developed models and all their terms were proven by statistic tests. A developed second-order polynomial equation showed that there was a complex interaction among the feedstock mass, the temperature, and the time, affecting pyrolysis oil yield. The developed multi-linear model showed that both the temperature and the time influenced the yield of solid residue, while the mass of the raw material did not have such an influence. The effect of the feedstock mass far exceeded the influence of the time and the mass on the liquid yield in the fixed-bed pyrolysis of plastic waste. This influence could be explained by studying the temperature change in the bottom and middle parts of the fixed-bed reactor. The recorded temperature diagrams showed that, with greater masses of feedstock, local overheating occurred in the middle part of the reactor, which led to the overcracking of volatile products and, consequently, an increased formation of non-condensable gases, thus reducing the yield of pyrolytic oil.

In the near future, our efforts will be focused on proving how this advanced control loop leads to a significantly higher liquid yield, with negligible overheating, i.e., with a uniform reactor temperature field. In this research, we used polypropylene only as a model sample, but other plastics, plastic mixtures, or plastic waste can be investigated by the method used. Namely, the observed findings would be the same for all other types of plastic materials because we have proven that the thermal behavior of the system is dominantly influenced by the mass of the raw material in combination with the developed temperature control system and the associated heating of the system.

## Figures and Tables

**Table 1:** Design of the experiment (Box–Behnken design).

	Factor 1	Factor 2	Factor 3	Response 1	Response 2
Run	A: Temperature	B: Mass	C: Time	Liquid Yield	Solid Residue
	°C	g	Min	%	%
1	475	100	60	56.43	6.88
2	500	100	45	41.85	1.93
3	450	50	60	62.82	23.26
4	450	150	60	46.02	20.68
5	500	150	60	40.73	1.96
6	475	50	75	66.54	3.76
7	475	150	75	47.17	1.81
8	475	100	60	58.95	6.09
9	475	50	45	46.8	36.28
10	450	100	75	55.72	25.94
11	450	100	45	39.75	44.89
12	475	100	60	58.34	5.94
13	475	150	45	41.49	8.11
14	500	50	60	60.18	1.42
15	500	100	75	41.15	1.6
16	475	100	60	56.31	6.58

**Table 2:** Fit summary statistics.

Response	Source	Sequential *p*-Value	Lack-of-Fit *p*-Value	Adjusted R^2^	Predicted R^2^	
1 Liquid yield	Linear	0.0109	0.0085	0.4898	0.2655	
	2FI	0.4536	0.0073	0.4843	−0.0773	
	Quadratic	0.0021	0.0877	0.9231	0.5707	Suggested
2 Solid residue	Linear	8.15	0.7156	0.6445	0.4479	Suggested
	2FI	7.72	0.8087	0.6812	0.2079	
	Quadratic	6.27	0.9157	0.7893	−0.3458	

**Table 3:** Adopted factors’ coding law.

Factor	Name	Units	Minimum	Maximum	Coded Low	Coded High	Mean
A	Temperature	deg C	450.00	500.00	−1 ↔ 450.00	+1 ↔ 500.00	475.00
B	Mass	g	50.00	150.00	−1 ↔ 50.00	+1 ↔ 150.00	100.00
C	Time	min	45.00	75.00	−1 ↔ 45.00	+1 ↔ 75.00	60.00

**Table 4:** ANOVA for the selected model (the factors are coded).

	Source	Sum of Squares	df	Mean Square	F-Value	*p*-Value
Response 1 Liquid yield	Model	1181.74	7	168.82	33.79	<0.0001
	A-Temperature	52.02	1	52.02	10.41	0.0121
	B-Mass	464.06	1	464.06	92.88	<0.0001
	C-Time	206.96	1	206.96	41.42	0.0002
	AC	69.47	1	69.47	13.90	0.0058
	BC	49.42	1	49.42	9.89	0.0137
	A^2^	119.96	1	119.96	24.01	0.0012
	C^2^	219.85	1	219.85	44.00	0.0002
	Residual	39.97	8	5.00		
	Lack of Fit	34.60	5	6.92	3.87	0.1474
Response 2 Solid residue	Model	1876.17	2	938.09	13.17	0.0007
	A-Temperature	1454.22	1	1454.22	20.41	0.0006
	C-Time	421.95	1	421.95	5.92	0.0301
	Residual	926.32	13	71.26		

**Table 5:** General-fit statistics.

Response 1 Liquid yield	Std. Dev.	2.24	R^2^	0.9673
	Mean	51.27	Adjusted R^2^	0.9387
	C.V. %	4.36	Predicted R^2^	0.7716
			Adeq Precision	17.9664
Response 2 Solid residue	Std. Dev.	8.44	R^2^	0.6695
	Mean	12.32	Adjusted R^2^	0.6186
	C.V. %	68.51	Predicted R^2^	0.4514
			Adeq Precision	11.3510

## Data Availability

Data are contained within this article.

## References

[1] Dai L., Zhou N., Lv Y., Cheng Y., Wang Y., Liu Y., Cobb K., Chen P., Lei H., Ruan R. (2022). Pyrolysis Technology for Plastic Waste Recycling: A State-of-the-Art Review. Prog. Energy Combust. Sci..

[2] Miandad R., Rehan M., Barakat M.A., Aburiazaiza A.S., Khan H., Ismail I.M.I., Dhavamani J., Gardy J., Hassanpour A., Nizami A.-S. (2019). Catalytic Pyrolysis of Plastic Waste: Moving Toward Pyrolysis Based Biorefineries. Front. Energy Res..

[3] Papuga S., Djurdjevic M., Ciccioli A., Vecchio Ciprioti S. (2023). Catalytic Pyrolysis of Plastic Waste and Molecular Symmetry Effects: A Review. Symmetry.

[4] Vouvoudi E.C., Rousi A.T., Achilias D.S. (2023). Effect of the catalyst type on pyrolysis products distribution of polymer blends simulating plastics contained in waste electric and electronic equipment. Sustain. Chem. Pharm..

[5] Gebre S.H., Sendeku M.G., Bahri M. (2021). Recent Trends in the Pyrolysis of Non-Degradable Waste Plastics. Chem. Open.

[6] Gulab H., Jan M.R., Shah J., Manos G. (2010). Plastic catalytic pyrolysis to fuels as tertiary polymer recycling method: Effect of process conditions. J. Environ. Sci. Health Part A.

[7] Dong Z., Chen W., Xu K., Liu Y., Wu J., Zhang F. (2022). Understanding the Structure–Activity Relationships in Catalytic Conversion of Polyolefin Plastics by Zeolite-Based Catalysts: A Critical Review. ACS Catal..

[8] Mondal B.K., Guha F., Abser M.N. (2023). Sol-gel derived Ti-doped mesoporous silica–alumina: An efficient catalyst to recover energy sources from environmental hazard waste plastics. J. Therm. Anal. Calorim..

[9] Serra A.C.S., Milato J.V., Faillace J.G., Calderari M.R.C.M. (2023). Reviewing the use of zeolites and clay based catalysts for pyrolysis of plastics and oil fractions. Braz. J. Chem. Eng..

[10] Fadillah G., Fatimah I., Sahroni I., Musawwa M.M., Mahlia T.M.I., Muraza O. (2021). Recent Progress in Low Cost Catalysts for Pyrolysis Plastic Waste to Fuels. Catalysts.

[11] Al-Salem S.M., Antelava A., Constantinou A., Manos G., Dutta A. (2017). A review on thermal and catalytic pyrolysis of plastic solid waste (PSW). J. Environ. Manag..

[12] Miandad R., Barakat M.A., Aburiazaiza A.S., Rehan M., Nizami A.S. (2016). Catalytic pyrolysis of plastic waste: A Review. Process Saf. Environ. Prot..

[13] Maqsood T., Dai J., Zhang Y., Guang M., Li B. (2021). Pyrolysis of plastic species: A review of resources and products. J. Anal. Appl. Pyrolysis.

[14] Ahmad I., Khan M.I., Khan H., Ishaq M., Tariq R., Gul K., Ahmad W. (2015). Pyrolysis Study of Polypropylene and Polyethylene Into Premium Oil Products. Int. J. Green Energy.

[15] Uebe J., Kryzevicius Z., Majauskiene R., Dulevicius M., Kosychova L., Zukauskaite A. (2022). Use of polypropylene pyrolysis oil in alternative fuel production. Waste Manag. Res..

[16] Jaafar Y., Abdelouahed L., Hage R.E., Samrani A.E., Taouk B. (2022). Pyrolysis of common plastics and their mixtures to produce valuable petroleum-like products. Polym. Degrad. Stab..

[17] Abbas-Abadi M.S., Haghighi M.N., Yeganeh H., McDonald A.G. (2014). Evaluation of pyrolysis process parameters on polypropylene degradation products. J. Anal. Appl. Pyrolysis.

[18] Achilias D.S. (2008). Recycling techniques of polyolefins from plastic wastes. Glob. NEST J..

[19] Aisien E.T., Otuya I.C., Aisien F.A. (2021). Thermal and Catalytic Pyrolysis of Waste Polypropylene Plastic Using Spent FCC Catalyst. Environ. Technol. Innov..

[20] Vijayakumar A., Sebastian J. (2018). Pyrolysis process to produce fuel from different types of plastic: A review. IOP Conf. Ser. Mater. Sci. Eng..

[21] Abdullah N.A., Novianti A., Hakim I.I., Putra N., Koestoer R.A. (2018). Influence of Temperature on Conversion of Plastics Waste (Polystyrene) to Liquid Oil Using Pyrolysis Process. IOP Conf. Ser. Earth Environ. Sci..

[22] Anuar Sharuddin S.D., Abnisa F., Wan Daud W.M.A., Aroua M.K. (2016). A Review on Pyrolysis of Plastic Wastes. Energy Convers. Manag..

[23] Đurđević M., Papuga S., Kolundžija A. (2024). Analysis of the thermal behavior of a fixed bed reactor during the pyrolysis process. Hem. Ind.

[24] Maafa I. (2021). Pyrolysis of Polystyrene Waste: A Review. Polymers.

[25] Papuga S., Gvero P., Vukic L. (2016). Temperature and Time Influence on the Waste Plastics Pyrolysis in the Fixed Bed Reactor. Therm. Sci..

[26] Esposito L., Cafiero L., De Angelis D., Tuffi R., Vecchio Ciprioti S. (2020). Valorization of the plastic residue from a WEEE treatment plant by pyrolysis. Waste Manag..

[27] Borsella E., Aguado R., De Stefanis A., Olazar M. (2018). Comparison of catalytic performance of an iron-alumina pillared montmorillonite and HZSM-5 zeolite on a spouted bed reactor. J. Anal. Appl. Pyrolysis.

[28] Cocchi M., Angelis D.D., Mazzeo L., Nardozi P., Piemonte V., Tuffi R., Vecchio Ciprioti S. (2020). Catalytic Pyrolysis of a Residual Plastic Waste Using Zeolites Produced by Coal Fly Ash. Catalysts.

[29] Khazaal R.M., Abdulaaima D.A. (2023). Valuable oil recovery from plastic wastes via pressurized thermal and catalytic pyrolysis. Energy Convers. Manag. X.

[30] Han H.-Z., Li B.-X., Wu H., Shao W. (2015). Multi-Objective Shape Optimization of Double Pipe Heat Exchanger with Inner Corrugated Tube Using RSM Method. Int. J. Therm. Sci..

[31] Amran M., Salmah S., Sanusi M., Yuhazri M., Mohamad N., Azam M.A., Abdullah Z., Mohamad E. (2014). Surface Roughness Optimization in Drilling Process Using Response Surface Method (RSM). J. Teknol..

[32] Bashir M.J.K., Abu Amr S.S., Aziz S.Q., Aun N.C., Sethupathi S. (2015). Wastewater Treatment Processes Optimization Using Response Surface Methodology (RSM) Compared with Conventional Methods: Review and Comparative Study. Middle-East J. Sci. Res..

[33] Said K.A.M., Amin M.A.M. (2016). Overview on the Response Surface Methodology (RSM) in Extraction Processes. J. Appl. Sci. Process. Eng..

[34] Dutta N., Mondal P., Gupta A. (2022). Optimization of Process Parameters Using Response Surface Methodology for Maximum Liquid Yield during Thermal Pyrolysis of Blend of Virgin and Waste High-Density Polyethylene. J. Mater. Cycles Waste Manag..

[35] Kumar S., Singh R.K. (2014). Optimization of Process Parameters by Response Surface Methodology (RSM) for Catalytic Pyrolysis of Waste High-Density Polyethylene to Liquid Fuel. J. Environ. Chem. Eng..

[36] Mo Y., Zhao L., Wang Z., Chen C.-L., Tan G.-Y.A., Wang J.-Y. (2014). Enhanced Styrene Recovery from Waste Polystyrene Pyrolysis Using Response Surface Methodology Coupled with Box–Behnken Design. Waste Manag..

[37] Pinto F., Paradela F., Gulyurtlu I., Ramos A.M. (2013). Prediction of Liquid Yields from the Pyrolysis of Waste Mixtures Using Response Surface Methodology. Fuel Process. Technol..

[38] Selvaganapathy T., Muthuvelayudham R., Jayakumar M. (2020). Process Parameter Optimization Study on Thermolytic Polystyrene Liquid Fuel Using Response Surface Methodology (RSM). Mater. Today Proc..

[39] Faisal F., Rasul M.G., Chowdhury A.A., Jahirul M.I. (2024). Optimisation of Process Parameters to Maximise the Oil Yield from Pyrolysis of Mixed Waste Plastics. Sustainability.

[40] Ore O.T., Adebiyi F.M. (2024). Process modelling of waste tyre pyrolysis for gas production using response surface methodology. Unconv. Resour..

[41] Thonglhueng N., Sirisangsawang R., Sukpancharoen S., Phetyim N. (2022). Optimization of iodine number of carbon black obtained from waste tire pyrolysis plant via response surface methodology. Heliyon.

[42] Quesada L., Pérez A., Godoy V., Peula F.J., Calero M., Blázquez G. (2019). Optimization of the pyrolysis process of a plastic waste to obtain a liquid fuel using different mathematical models. Energy Conv. Manag..

[43] Wirawan R., Farizal P. Plastic waste pyrolysis optimization to produce fuel grade using factorial design. Proceedings of the 4th International Conference on Energy, Environment, Epidemiology and Information System (ICENIS 2019), E3S Web Conference.

[44] Gonzalez-Aguilar A.M., Cabrera-Madera V.P., Vera-Rozo J.R., Riesco-Ávila J.M. (2022). Effects of Heating Rate and Temperature on the Thermal Pyrolysis of Expanded Polystyrene Post-Industrial Waste. Polymers.

[45] Irfan M., Nabi R.A.U., Hussain H., Naz M.Y., Shukrullah S., Khawaja H.A., Rahman S., Ghanim A.A.J., Kruszelnicka I., Ginter-Kramarczyk D. (2020). Response Surface Methodology Analysis of Pyrolysis Reaction Rate Constants for Predicting Efficient Conversion of Bulk Plastic Waste into Oil and Gaseous Fuel. Energies.

[46] Xie S., Kumagai S., Kameda T., Saito Y., Yoshioka T. (2021). Prediction of pyrolyzate yields by response surface methodology: A case study of cellulose and polyethylene co-pyrolysis. Bioresour. Technol..

[47] Papuga S., Djurdjevic M., Tomović G., Vecchio Ciprioti S. (2023). Pyrolysis of Tyre Waste in a Fixed-Bed Reactor. Symmetry.

[48] Kremer I., Tomić T., Katančić Z., Erceg M., Papuga S., Vuković J.P., Schneider D.R. (2021). Catalytic Pyrolysis of Mechanically Non-Recyclable Waste Plastics Mixture: Kinetics and Pyrolysis in Laboratory-Scale Reactor. J. Environ. Manag..

[49] Hartulistiyoso E., Sigiro F., Yulianto M. (2015). Temperature Distribution of the Plastics Pyrolysis Process to Produce Fuel at 450 °C. Procedia Env. Sci..

[50] Lee C.G., Cho Y.J., Song P.S., Kang Y., Kim J.S., Choi M.J. (2003). Effects of temperature distribution on the catalytic pyrolysis of polystyrene waste in a swirling fluidized-bed reactor. Catal Today.

[51] Cahyono M.S., Fenti U.I. (2017). Influence of Heating Rate and Temperature on the Yield and Properties of Pyrolysis Oil Obtained from Waste Plastic Bag. Conserve J. Energy Environ. Stud..

